# Documenting Disease in the Undocumented Migrants: A Case Report of Chronic Kidney Disease of Unknown Origin in a Central American Migrant

**DOI:** 10.7759/cureus.24566

**Published:** 2022-04-28

**Authors:** Adityabikram Singh, Brittany M Zaita, Isha Gupta, Gurjinder Kaur

**Affiliations:** 1 Health Sciences, Rutgers University New Jersey Medical School, Newark, USA; 2 Health Sciences, Touro College of Osteopathic Medicine, Middletown, USA; 3 Nephrology, Middletown Medical, Middletown, USA; 4 Nephrology, Internal Medicine, Middletown Medical, Middletown, USA; 5 Physiology, Touro College of Osteopathic Medicine, Middletown, USA

**Keywords:** glomerulosclerosis, mesoamerican nephropathy, kidney biopsy, nephrotoxin, kidney replacement therapy, undocumented migrants, tubulointerstitial fibrosis, glomerulonephropathy, chronic kidney disease of unknown aetiology

## Abstract

Mesoamerican nephropathy (MeN) or chronic kidney disease of unknown origin (CKDu) is a rising epidemic in hotspot regions of El Salvador and Nicaragua. MeN is often defined in patients who exhibit a clinically reduced estimated glomerular filtration rate (eGFR) but lack a defining etiology such as diabetes or hypertension. A multitude of risk factors for MeN have been identified, including physical labor demands in a hot climate, exposure to pesticides, and poverty. Additionally, social determinants such as limited access to health care and the cost of disease burden often contribute to overall poor prognosis and progression of the disease.

We present a case of a 39-year-old male with a past medical history of gout who presented to the emergency room with abdominal pain radiating to the flanks and bilateral great toe pain. Social history revealed the patient recently moved to the United States from Central America (Nicaragua), was unemployed, and did not have health insurance. Prior to the presentation, the patient admitted he was not compliant with his gout medications for about one month. The symptoms first began two to three weeks prior to his evaluation in the emergency department; the patient also endorsed decreased oral intake during this time period. He was noted to have abnormally elevated creatinine along with elevated uric acid levels, low potassium and magnesium levels. Abdominal imaging revealed nephrolithiasis without hydronephrosis. Initial differentials included acute kidney injury (AKI) from dehydration, non-steroidal anti-inflammatory drug (NSAID) induced nephropathy, and uric acid nephropathy. This patient was eventually found to have a biopsy-proven findings of CKDu. We want to highlight the need to keep MeN high in the differential with a low threshold to perform a renal biopsy for accurate diagnosis and management of the disease, especially in the rising immigrant population in the United States.

## Introduction

Chronic kidney disease (CKD) affects roughly 7.2% of the population of the United States, and in recent years seems to be increasing in prevalence [1]. Comparatively, in hotspot regions of El Salvador and Nicaragua, the prevalence of CKD is much higher, where studies have estimated the disease in 17% and 42% of the male population respectively [2]. Of note is a subset of patients in these regions who show clinically reduced eGFR but often lack a defining etiology such as genetic kidney disease, infection, or chronic metabolic disease. These patients are classified as having chronic kidney disease of unknown origin (CKDu) or mesoamerican nephropathy (MeN). A multitude of risk factors for MeN have been identified, most importantly physical labor demands in a hot climate, exposure to pesticides, and poverty [2]. 

A study by Sanchez Polo et al. identifies a timeline of the origin of the epidemic of MeN in Central America. In 2002, the first clinical description of MeN in El Salvador was postulated to be caused by toxic environmental or heavy metal exposures. In 2003, similar cases arose in Mexico, Guatemala, El Salvador, and Honduras. In 2005, Costa Rica reported their first patient with MeN; field studies were designed to examine the prevalence of the disease, and new theories suggested that MeN was linked to a genetic cause or certain occupational exposures. In 2009, Nicaragua reported its first case of MeN. Finally, in 2013, the Pan American Health Organization (PAHO) officially recognized MeN as an epidemic, and kidney biopsies began being performed on patients in El Salvador [3].

Though a true defining etiology for MeN is yet to be identified, examination of patients in endemic areas has provided great clinical insight into the disease process and major risk factors associated with disease presentation. García-Trabanino et al. studied cases of MeN in the Baja Lempo region of El Salvador and found that MeN affects males: females in a 9:1 ratio, 84.7% of affected males worked in agriculture, and 66% of cases had no history of diabetes or hypertension [4]. Ferguson et al. examined cases in Southwestern Nicaragua and found that working in the sugarcane industry was the strongest associated risk factor with confirmed CKD cases [5]. It is well understood that agricultural labor creates an environment of chronic heat stress and dehydration which may impact kidney function over time. The theory of occupational exposure to pesticides and their metabolites arose due to the presence of disease in women who stayed at home, and the lack of disease in other populations of workers under heat stress [6]. Lastly, chronic NSAID use is considered to be a potential cofactor in the pathogenesis of MeN due to findings of glomerular ischemia with minor vascular involvement on kidney biopsy [2]. Correa-Rotter et al. also support that NSAIDs can decrease renal perfusion, which can accelerate kidney damage, especially in a dehydrated state [7].

Given the severity and high mortality rate of MeN, it is important to highlight how these patients may go undiagnosed, especially among immigrant populations in the United States. Additionally, though patients exhibit a clinically reduced eGFR, there is often a high threshold to performing kidney biopsy which is ultimately diagnostic for patients with MeN. To emphasize this, Ferguson et al. state that kidney biopsies often show chronic tubulointerstitial damage, with or without evidence of glomerular ischemia or glomerulosclerosis [5]. Also of note, Elinder et al. support that the histopathologic changes of CKDu may be more severe outside of Mesoamerica despite a less degree of heat exposure and strenuous work [2]. Finally, the healthcare providers must consider the overwhelming financial burden for the undiagnosed and untreated MeN, as it will eventually lead to end stage renal disease (ESRD) and the need for renal replacement therapy (RRT) through either dialysis or renal transplant evaluation.

## Case presentation

Our patient is a 39-year-old Spanish-speaking male whose only known medical condition was gout treated with colchicine, who presented to the hospital with complaints of flank pain and bilateral toe pain. The flank pain was present for two weeks duration and was preceded by toe swelling for three weeks duration. He denied any nausea, vomiting, fevers, or chills. He had recently moved to the United States (late 2021) and reported nonadherence to his medical regimen of colchicine for the treatment of gout. He denied any known history of hypertension, diabetes mellitus, obesity, or kidney stones and endorsed the use of Toradol and other NSAIDs at least nine days per month for pain relief for a period of at least 10 years. He denied any knowledge of the renal disease, but also had not been evaluated by a physician for a period of years. The patient denied any family history of renal or metabolic disease, tobacco use, alcohol use, or illicit drug use. The patient also denied any known exposure to pesticides. Occupational history was not available. 

**Figure 1:**
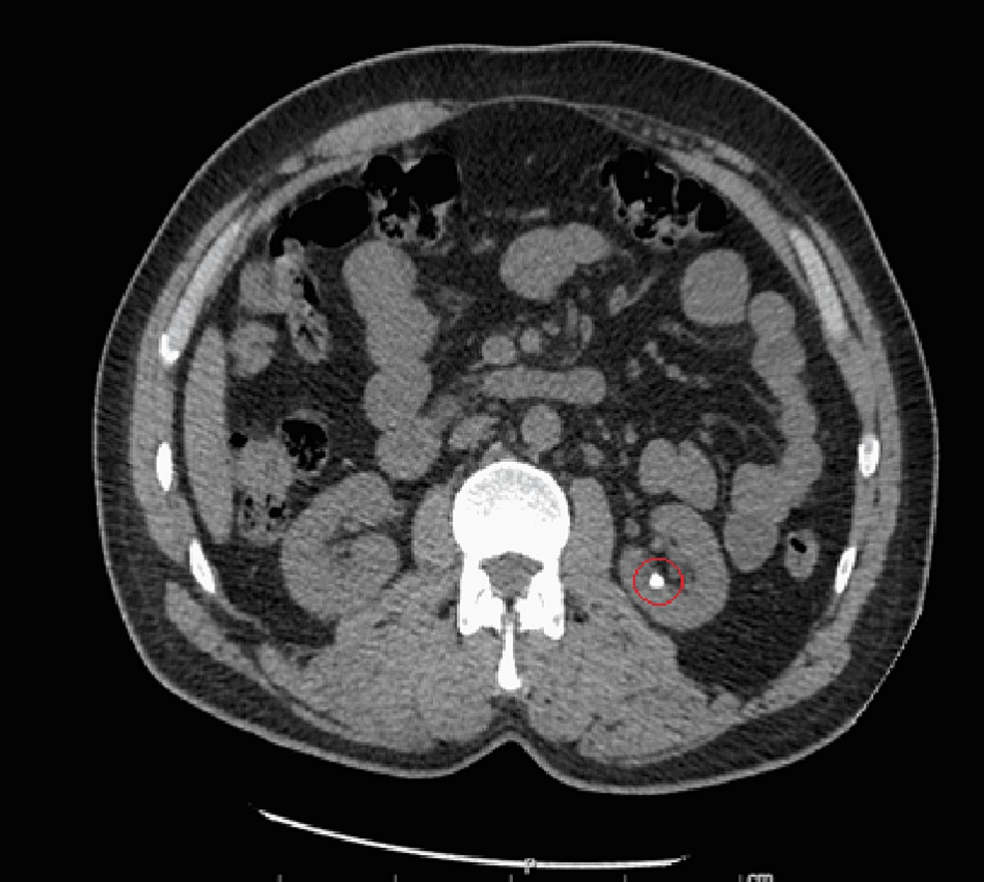
Axial view of CT abdomen pelvis without IV contrast. Red circle marking 6 mm non-obstructing left renal stone.

**Figure 2:**
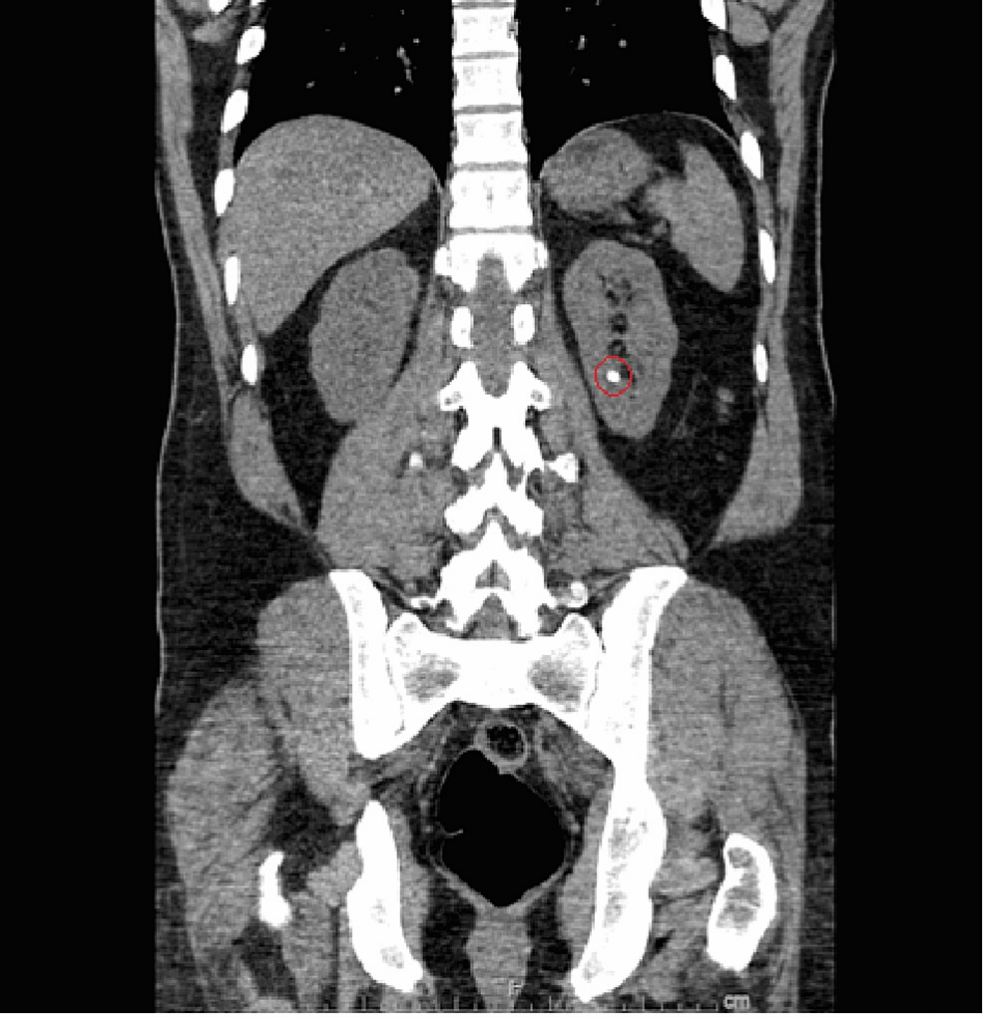
Coronal view of CT abdomen pelvis without IV contrast. Red circle marking 6 mm non-obstructing left renal stone.

**Figure 3:**
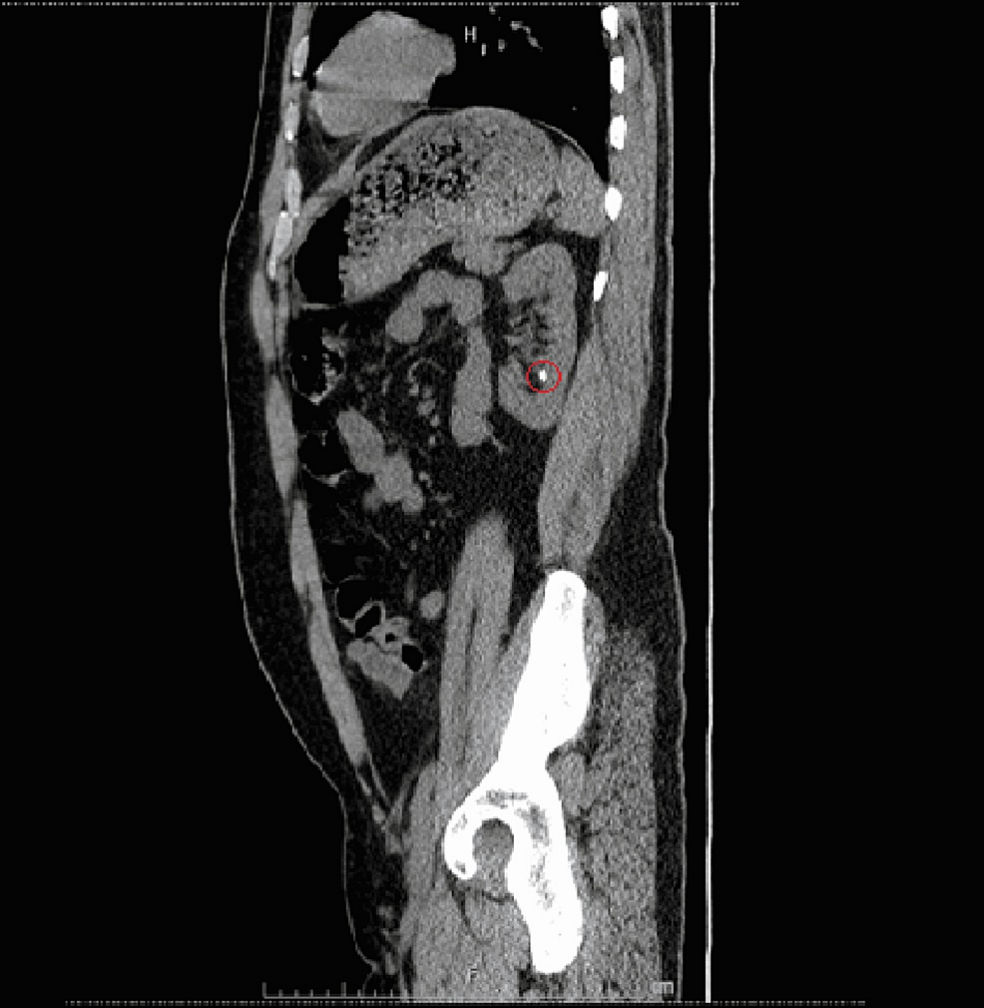
Sagittal view of CT abdomen pelvis without IV contrast. Red circle marking 6 mm non-obstructing left renal stone.

**Figure 4:**
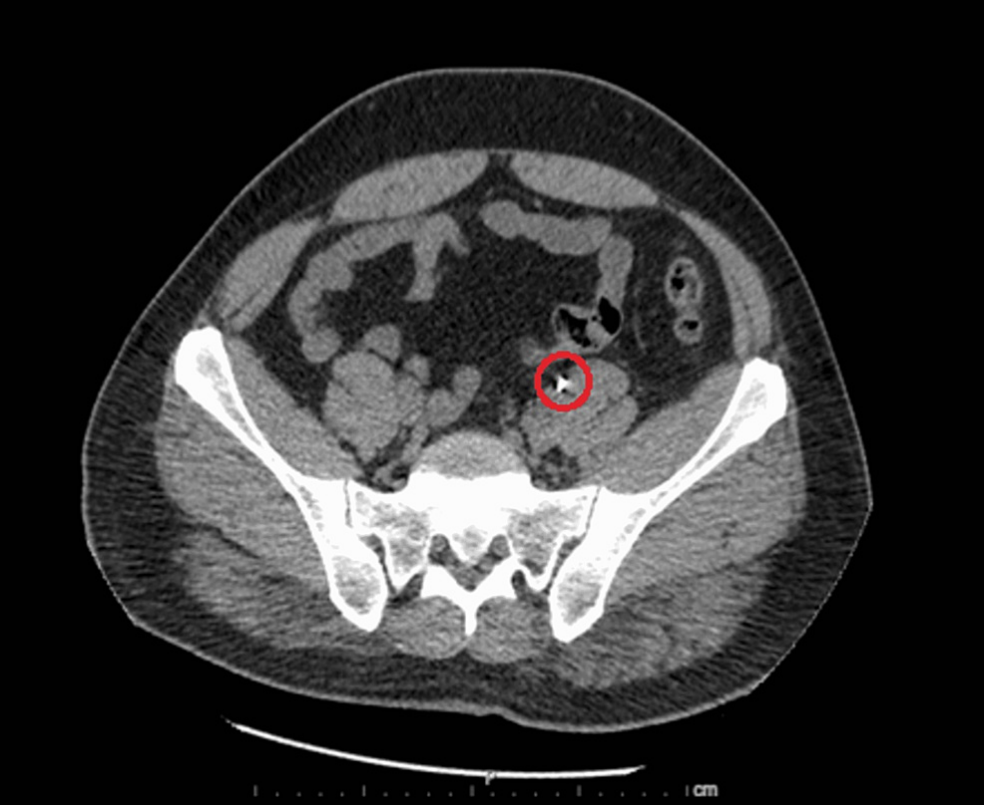
Axial view of CT abdomen pelvis without IV contrast. Red circle marking 9 mm left pelvic calcification, possible distal ureteral stone without hydronephrosis or hydroureter.

At the time of admission, the patient had a stable temperature, heart rate within normal limits and without rhythm abnormalities, mildly elevated blood pressure to 137/87 mmHg, stable respiration, oxygen saturation of 97%, and a body mass index (BMI) of 28.62 kg/m2. Physical exam was unremarkable aside from generalized abdominal tenderness and swelling of bilateral great toes (right greater than left). No flank tenderness was noted on the physical exam. Initial complete blood count (CBC) revealed mild anemia with leukocytosis and reactive thrombocytosis. The patient also had electrolyte abnormalities including hypokalemia, hypochloremia, and hyponatremia. His serum creatinine was elevated to 2.59 (with eGFR ~ 27.7 mL/min/1.53 m2); which later improved to 2.3 (eGFR ~ 31.8 mL/min/1.53 m2) with IV hydration. The remainder of the initial workup revealed elevated uric acid level to 10.2 (normal 3.4-7.0 mg/dL), hypomagnesemia with Mg ~ 1.3 mg/dL (normal 1.8-2.5 mg/dL), and normal urinalysis with the exception of trace protein. Urine electrolytes were ordered to calculate fractional excretion of sodium, which was less than 1%, traditionally indicative of pre-renal pathology. CT scan of the abdomen and pelvis was performed for further evaluation, which revealed a 6 mm non-obstructing left renal stone. A 9 mm left pelvic calcification that was possibly a distal left ureteral stone without hydronephrosis or hydroureter was noted as well (Figures 1-4).

Initial differential at this stage included acute kidney injury (AKI) from dehydration, as he admitted to poor oral intake since the pain started. This was thought to be exacerbated by the use of NSAIDs and his concurrent flare-up of gout. The patient was evaluated by urology and underwent cystoscopy, left retrograde pyelogram, and left ureteroscopy, which found no obstructive stones or hydronephrosis. Ureteral stenting was not indicated. With fluid resuscitation and management of gout flare-up with steroids and colchicine, the patient's renal function showed minimal improvement with creatinine nadir of 2.12 mg/dL (eGFR < 35 mL/min/1.53 m2). At this time further workup for underlying kidney disease was done, with results of admission blood work (Table 1) and results of common causes of nephropathy detailed below (Table 2). 

**Table 1:** Admission blood work.

Test	Result (normal value)
Complete blood count	
White blood cells	11.7 (4.3 - 10.6 10^3/µL )
Red blood cells	4.10 (4.30 - 5.80 10^6/µL)
Hemoglobin	11.3 (13.3 - 17.0 g/dL)
Hematocrit	33.0 (40.3 - 50.3 %)
Mean corpuscular volume	80.5 (81.4 - 99.0 fL)
Mean corpuscular hemoglobin	27.6 (26.8 - 32.9 pg)
Mean corpuscular hemoglobin concentration	34.2 (31.4 - 34.9 g/dL)
Red cell distribution width	12.8 (11.6 - 14.9 %)
Platelets	455 (132 - 337 10^3/µL )
Basic metabolic panel	
Sodium	135 (136 - 144 mEq/L)
Potassium	2.7 (3.5 - 5.1 mEq/L)
Chloride	92 (101 - 111 mEq/L)
CO2	29 (22 - 32 mEq/L)
Blood urea nitrogen	35 (8 - 20 mg/dL)
Glucose	107 (70 - 110 mg/dL)
Calcium	9.6 (8.5 - 10.1 mg/dL)
Creatinine	2.59 (0.55 - 1.02 mg/dL)
Liver function panel	
Albumin	3.1 (3.5 - 4.8 g/dL)
Total protein	8.1 (6.4 - 8.3 g/dL)
Total bilirubin	0.3 (0.3 - 1.2 mg/dL)
Bilirubin, direct	0.1 (0.1 - 0.5 mg/dL)
Bilirubin, indirect	0.2 (0.2 - 0.7 mg/dl)
Alkaline phosphatase	84 (45-117 U/L)
Aspartate transaminase	32 (15-41 U/L)
Alanine transaminase	44 (17-63 U/L)

**Table 2:** Results of inpatient workup for common causes of nephropathy. PCR, polymerase chain reaction; ANA, antinuclear antibodies; RF, rheumatoid factor; IgA, immunoglobulin A

Test	Result (Normal range)
Human immunodeficiency virus PCR	Negative
Lupus panel	Mildly reactive
ANA	1:40 (<1:4)
RF	Negative
Complement component 3 (C3) level	138 (75-175 mg/dL)
Complement component 4 (C4) level	22 (14-40 mg/dL)
Anti-neutrophil cytoplasmic antibodies (C and P)	Negative
Hepatitis panel	Negative
anti–Sjögren's-syndrome type A and B autoantibodies (SS-A/SS-B)	Negative
Reticulin IgA screen	Negative
Anti-Smith antibody (SM-AB)	Negative
Antinuclear ribonucleoprotein antibodies (anti-RNP)	Negative
Topoisomerase 1 autoantibodies (SCL-70 AB)	Negative
Anti-ribosomal protein (Anti-Rib-P) antibody	Negative
Anti-actin (smooth muscle) antibody IgG levels	20 (<20 negative)

**Figure 5:**
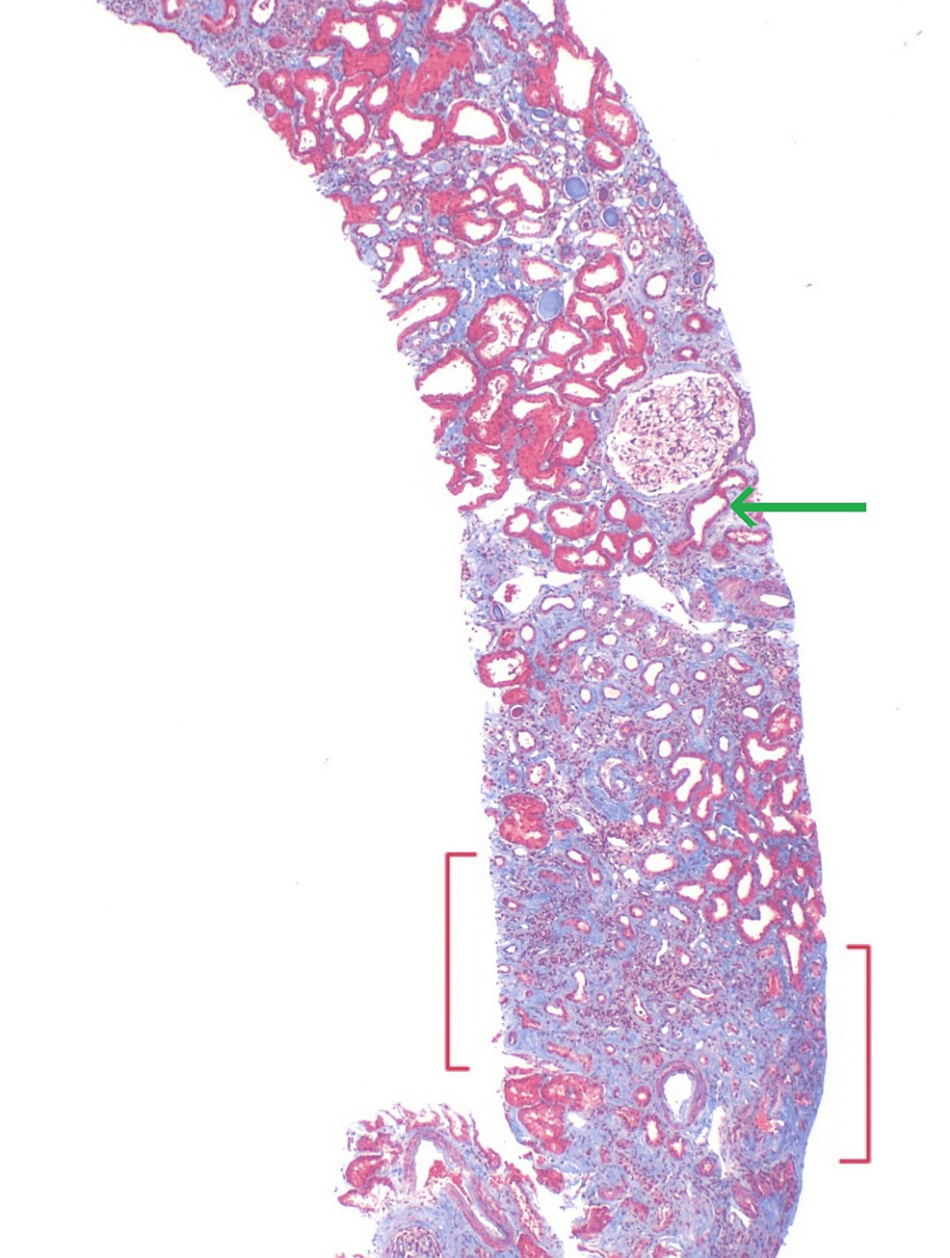
Renal biopsy tissue core measuring 17 mm, trichrome stain. Denudation of tubular epithelium marked with green arrow. Areas of cortical scarring identified between the red brackets.

**Figure 6:**
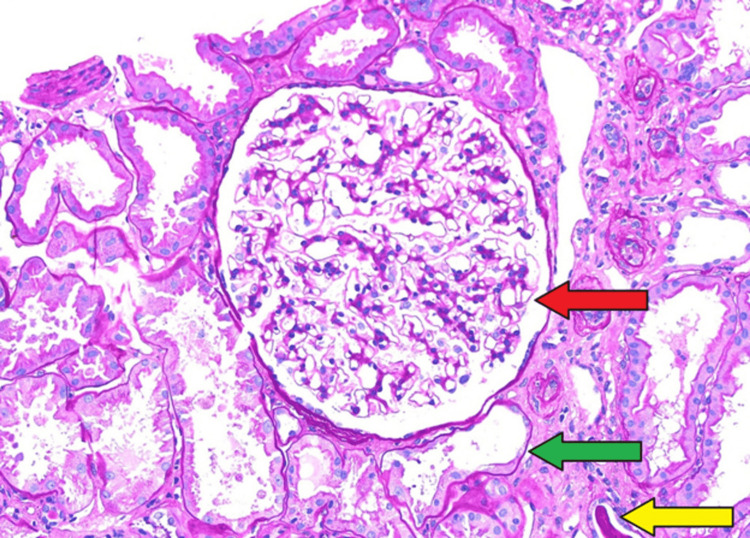
High power image of glomerulus and surrounding structures, PAS stain. Glomerulomegaly is marked with a red arrow. Irregular thickening and thinning of tubular basement membrane marked with green arrow. Hyaline cast marked with a yellow arrow. PAS, periodic acid-Schiff

Following an unremarkable workup, the patient consented to a percutaneous renal biopsy. Renal biopsy at the Department of Renal Pathology, Columbia University in New York, revealed evidence of moderate chronic tubulointerstitial nephropathy (CTIN) and secondary focal global glomerulosclerosis with glomerulomegaly. He had mild arterio- and arteriolosclerosis. Findings were thought to be suggestive of an endemic form of CTIN (i.e., MeN) (Figures 5-6).

## Discussion

The CKDu or MeN is a fast-growing endemic in Central America and a concerning clinical entity for physicians in the United States due to increasing rates of immigration from the region. Many theories have been proposed as to the etiology of the condition which includes genetic predisposition combined with exposure to heat stress, dehydration, environmental toxins, and heavy metal exposure. The predilection of this disease for agricultural workers is especially shocking. A paper by Rajapaksha, 2021 shows that there are significant interactions between key renal enzymes and commonly used pesticides and their metabolites [8]. Specifically, this study found that dysfunction caused by quinalphos and chlorpyrifos binding renal 5' adenosine monophosphate-activated protein kinase (AMPK) resulted in fibrosis of interstitial tubules, a common finding in kidney biopsies of MeN patients [5]. Another affected renal enzyme was protein kinase C (PKC), which when bound by pesticides (fenthion, fenamiphos, and imidacloprid), and their metabolites resulted in increased reactive oxidative species formation in the renal parenchyma. Elinder et al. explain how low altitude regions are at a greater risk of groundwater contamination when compared to high altitude regions [2]. Further evidence for this theory is seen in the markedly reduced prevalence of CKDu in coffee farmers in the high-altitude regions of Central America, where rates of agrochemical exposure are similar, but groundwater contamination is negligible [5]. Another point from the study is the reduced rates of heat stress in high-altitude workers. Compared to the cooler and drier climate in the mountains, the climate conditions in the coastal, low-lying regions of Central America predispose the workers there to higher levels of dehydration and heat stress.

Multiple studies also mentioned the importance of genetic predisposition in the development of disease as well as the use of NSAIDs in manual laborers as risk factors for CKDu. Workers with CKDu often had family members with metabolic diseases such as hypertension or diabetes [2-3, 5, 7]. Other studies found an increased rate of NSAID use in manual laborers, which as a known nephrotoxin, may exacerbate risk from other causative factors in agricultural workers [7]. De Broe and Vervaet, and Rajapaksha also identified other CKDu hotspots around the world in India, Egypt, Cuba, Bangladesh, Sri Lanka, and France [6, 8]. In each hotspot, the same common variables were present: agrochemicals, heat stress, groundwater contamination, and the population at risk was again largely male, agricultural workers. This study also found that schoolchildren and women in these areas had elevated concentrations of markers for tubular injuries such as neutrophil gelatinase-associated lipocalin and N-acetyl-D-glucosaminidase indicating early tubular damage in not just agricultural workers but the population at large in these hotspots [6].

Unsurprisingly, it is often those with the least resources who suffer the worst consequences of this disease. Low socioeconomic status is a global predictor of poor health outcomes, but that effect is exacerbated in patients with CKDu [9-10]. Lack of health follow-up can lead to rapid progression of this mysterious disease and preclude the patient from implementing early interventions that could have saved crucial kidney function. In rural regions of Central America, reduced availability of resources for the treatment of ESRD with RRT such as medical staff or dialysis centers means that a majority of the patients with this disease are dying of complications of kidney dysfunction without treatment. Sugarcane cutters, who are most frequently impacted, often start work at dawn and exceed Occupational Safety and Health Administration limitations of heat stress as early as 9:15 am [7]. Despite a recent investment of $15 million from the International Finance Corporation to Nicaraguan sugarcane companies for the development of strategies for combating the epidemic, no research or intervention has been authorized. The money has seemingly vanished with little reprise for workers who continue to endure poor working conditions and suffer from the disease [7]. For patients in the United States, the situation is also complicated. The often-undocumented status of migrants leaves them without many of the novel options for treatment of CKD that are available to the American public. Without health insurance or legal status these patients can neither afford to nor can they qualify for advanced RRT. While international organizations agree that the responsibility for addressing MeN lies with national and regional institutions in endemic areas, it is obvious that more needs to be done globally for this at-risk population.

Treatment plans for MeN consist mainly of supportive care and avoidance of further nephrotoxicity. Since the etiology of MeN is still undetermined, no exact therapeutic options are available or have been tested in patients with this disease. However, some general principles guiding the treatment of this disease are similar to those offered to patients with genetic or acquired CKD and include proper hydration, avoidance of nephrotoxic medications such as NSAIDs or herbal medications, the establishment of care with a nephrologist or primary care physician that can monitor disease progression closely. Preventative measures that are specific to the at-risk population include frequent rest breaks and reduction of heat exposure in the occupational setting, minimization of alcohol consumption, specifically home-brewed drinks, and the use of appropriate protective equipment in the application and handling of agrochemicals and heavy metals. In the case of our patient, special importance must be taken to treat his gout, a potentially injurious condition to the renal system, with medications that would not further exacerbate his disease [3].

Multiple studies show that pharmacological therapies such as angiotensin-converting enzyme inhibitors (ACEi) or angiotensin II receptor blockers (ARBs) are ineffective at treating MeN unless there is also an underlying metabolic condition such as diabetes or hypertension [2,3]. Lastly, when treating ESRD in patients with MeN, the treatment of last resort is RRT. While RRT may prolong life for patients with ESRD, it represents a huge cost to patients and healthcare systems alike as well as a significant reduction in quality of life from baseline. Despite 34.7% of patients receiving RRT, where treatment was mainly funded by government programs, 90.8% of patients died after 10 years (average age of death = 56.1 years) [4]. Due to undocumented status and lack of health insurance, patients with ESRD secondary to MeN do not qualify for kidney transplants and must use dialysis as their primary means of RRT. The lifetime costs of RRT can easily outweigh the cost of a single diagnostic study for this disease, which underscores the importance of prompt kidney biopsy and preventative measures in any patient suspected to have MeN.

 Some limitations, in this case, include lack of medical follow-up and low healthcare literacy in our patients. The patient had not seen a physician in many years. It is very likely that his condition developed when he was living and working in Nicaragua and was exacerbated by the stresses of hard labor. Without medical attention, this disease may have rapidly progressed and the lack of follow-up leaves us without a trace of the pathogenesis of his disease. Lack of family history is another limitation in this case; in the age of modern medicine, an investigation feels incomplete without a proper genetic workup. The fact that this condition arises in families and seems to be at least somewhat inherited hints at the presence of either a strong genetic or environmental component, which cannot be fully evaluated without a thorough family history and screen. Further environmental and enzymatic studies building upon the work of the De Broe and Rajapaksha studies are warranted to fully understand the biomechanics and pathogenesis of this disease.

This case is of critical importance due to the present social situation at the southern border of the United States and the country as a whole. Deteriorating socio-economic conditions in Central America, driven largely by political instability, illicit drug activities, and gang violence, have caused a seismic shift in-migration into the United States [11]. Diseases such as CKDu that were once endemic in Central America and other agricultural areas of the world now have a growing presence in our healthcare institutions. A growing need for the prevention and treatment of MeN exists and will need to be addressed in the years to come. Our team believes that discovering the origins of MeN and treating this disease is only one piece of the puzzle in understanding and alleviating the plight of Central American immigrants in the United States. This case highlights the need for a low threshold for kidney biopsy in any patient with suspected MeN. Since the treatment is mainly supportive and the economic and social costs of RRT are high, an ounce of prevention is truly worth a pound of cure in this case. Advising providers to implement preventative interventions and detailed care plans early in the hospital stay is another important strategy for limiting the progression of this disease. Patients in this at-risk population are less likely to follow up due to economic concerns and the measures described above can aid in reducing the burden of this disease on society. We hope that this report can bring awareness towards and help guide clinicians in the diagnosis and treatment of this condition.

## Conclusions

The epidemic of CKD of unknown origin is equally tragic from both a societal and a healthcare standpoint. It attacks one of the most vital organs in the body in one of the most vulnerable populations worldwide. Special care and attention must be given to this disease and the scenario given the present situation in Central America and resultant migration trends towards the United States. It is important that providers keep this differential in mind for young patients who may present from Central American countries with symptoms such as fever, polyuria, hyperuricemia, nocturia, and electrolyte abnormalities. Furthermore, it is equally crucial that providers maintain a low threshold for kidney biopsy as it is the only diagnostic measure for this disease in this population that is financially prohibited from seeking out health care in the United States. We hope this report can raise awareness towards this epidemic, inspire further research into the disease, and provide insight to clinicians who may encounter this disease in the future.
